# Synergistic Effect of Bupleuri Radix and Scutellariae Radix on Adipogenesis and AMP-Activated Protein Kinase: A Network Pharmacological Approach

**DOI:** 10.1155/2018/5269731

**Published:** 2018-08-23

**Authors:** Jueun Lee, Jinbong Park, Hyewon Park, Dong-Hyun Youn, Jaehoon Lee, Seokbeom Hong, Jae-Young Um

**Affiliations:** ^1^Department of Pharmacology, College of Korean Medicine, Kyung Hee University, Seoul, Republic of Korea; ^2^Basic Research Laboratory for Comorbidity Regulation, Comorbidity Research Institute, Kyung Hee University, Seoul, Republic of Korea; ^3^Department of Biological Sciences in Korean Medicine, Graduate School, Kyung Hee University, Seoul, Republic of Korea; ^4^Department of Korean Medicine, Graduate School, Kyung Hee University, Seoul, Republic of Korea

## Abstract

Obesity has become a major health threat in developed countries. However, current medications for obesity are limited because of their adverse effects. Interest in natural products for the treatment of obesity is thus rapidly growing. Korean medicine is characterized by the wide use of herbal formulas. However, the combination rule of herbal formulas in Korean medicine lacks experimental evidence. According to* Shennong's Classic of Materia Medica*, the earliest book of herbal medicine, Bupleuri Radix (BR) and Scutellariae Radix (SR) possess the Sangsoo relationship, which means they have synergistic features when used together. Therefore these two are frequently used together in prescriptions such as Sosiho-Tang. In this study, we used the network pharmacological method to predict the interaction between these two herbs and then investigated the effects of BR, SR, and their combination on obesity in 3T3-L1 adipocytes. BR, SR, and BR-SR mixture significantly decreased lipid accumulation and the expressions of two major adipogenic factors, peroxisome proliferator-activated receptor-gamma (PPAR*γ*) and CCAAT/enhancer-binding protein-alpha (C/EBP*α*), and their downstream genes,* Adipoq*,* aP2,* and* Lipin1* in 3T3-L1 cells. In addition, the BR-SR mixture had synergistic effects compared with BR or SR on inhibition of adipogenic-gene expressions. BR and SR also inhibited the protein expressions of PPAR*γ* and C/EBP*α*. Furthermore, the two extracts successfully activated AMP-activated protein kinase alpha (AMPK *α*), the key regulator of energy metabolism. When compared to those of BR or SR, the BR-SR mixture showed higher inhibition rates of PPAR*γ* and C/EBP*α*, along with higher activation rate of AMPK. These results indicate a new potential antiobese pharmacotherapy and also provide scientific evidence supporting the usage of herbal combinations instead of mixtures in Korean medicine.

## 1. Introduction

The epidemiology of obesity is constantly increasing, becoming a health threat all over the world. The World Health Organization has reported that over 1.4 billion adults worldwide are overweight (BMI ≥ 25 kg/m^2^) or obese (BMI ≥ 30 kg/m^2^) [1]. However, there are only 5 treatments (orlistat, lorcaserin, liraglutide, phentermine/topiramate, and bupropion/naltrexone) approved by the United States Food and Drug Administration. Despite their efficacies, adverse effects of these drugs lead to search for other options [2].

Obesity is caused by a simple process: when energy intake exceeds expenditure, the excess energy is saved in as a form of lipid. On the other hand, in the perspective of selecting treatments, there are more than pathways to resolve this metabolic syndrome. Pharmacological approach for obesity treatment can be subcategorized into two methods: inhibiting lipid accumulation or consuming accumulated lipids. Former method inhibits lipid accumulation in adipose tissues by decreasing appetite, lowering fat absorption, and inhibiting adipogenesis, and the latter is by increasing energy expenditure via fatty acid oxidation or brown adipose tissue- (BAT-) related thermogenesis [3]

Network pharmacology is a system biology-based methodology for pharmacological research [4, 5]. Network pharmacological research replaces the dominant paradigm of drug design based on “one gene, one drug, one disease,” into multitarget drugs that act on biological networks. These unique characteristics of network pharmacology expand the potential use of traditional herbal medicine including Korean medicine, Japanese Kampo medicine, and Traditional Chinese Medicine [6]. In this premise, as obesity is a multipathway-involved disease, and the multicomponent-multitarget herbal drugs may provide an answer to its treatment.

Bupleuri Radix (BR) and Scutellariae Radix (SR) are both components of Sosiho-Tang, a traditional Korean herbal formula which is originally used to treat chronic herbal diseases. The antiobese effect of Sosiho-Tang is reported in a study using 3T3-L1 adipocytes and high fat diet- (HFD-) induced obese C57BL/6J mice [7]. In the current study, Yoo et al. show Sosiho-Tang can inhibit adipogenesis by suppressing CCAAT/enhancer-binding protein-alpha (C/EBP*α*) and peroxisome proliferator-activated receptor-gamma (PPAR*γ*), two key regulators of adipogenesis* in vivo* and* in vitro*. Furthermore, other studies demonstrate that BR can attenuate obesity in rats by regulating adipogenic factors [8], while its active compound saikosaponin A inhibits inflammatory markers in adipocytes [9]. Several reports also demonstrate the antiobese and antiadipogenic effect of SR [10] and its active compounds such as baicalin [11–14], baicalein [15, 16], and wogonin [17–19].

Based on these studies, we expected that BR and SR are the major components of Sosiho-Tang responsible for antiobese effects. They are used for specific types of obesity patients after diagnosis by a Korean medicine practitioner, and, in addition, several experimental studies also report the possibility. According to* Shennong's Classic of Materia Medica*, a classic of oriental medicine introducing 365 kinds of herbs and their features [20], BR and SR are Sangsoo (synergistic) pairs. As a pair of Sangsoo (synergism), our hypothesis herein is that BR and SR may show synergistic effects in obesity-related mechanisms. Therefore, in this study, we evaluate the synergistic potential between BR and SR by the network pharmacological approach and assess their effects on obesity by investigating related mechanisms.

## 2. Materials and Methods

### 2.1. Reagents

3-Isobutylmethylxanthine (IBMX), dexamethasone (Dex), and Insulin were purchased from Sigma Chemicals (St Louis, MO, USA). Dulbecco's modified Eagle's medium (DMEM), penicillin-streptomycin, bovine serum (BS), and fetal bovine serum (FBS) were purchased from Gibco BRL (Grand Island, NY, USA).

### 2.2. Preparation of Water Extracts of BR and SR

BR (the root of Bupleurum falcatum Linne) and SR (the root of Scutellaria baicalensis Georgi), both originated from China, were purchased from Omniherb Co. (Daegu, Republic of Korea) identified by a specialist of herbs. 100 g of BR was extracted in 1000 ml of distilled water at 100°C for 4 h. 100 g of SR was extracted by the same method. Then, the water extracted BR and SR were freeze dried. The yields were 15.9% and 17.6% for BR and SR, respectively. The same amounts of BR and SR were mixed to prepare the BR-SR mixture.

### 2.3. Network Pharmacological Analysis

The herbal compound-target gene information was analyzed using the Traditional Chinese Medicine Systems Pharmacology Database (TCMSP). The major chemical components of SR and BR were collected and extracted from TCMSP. We collected each 99 and 147 compounds that encompassed the main components of SR and BR, taking into account the relevant reports. Then we selected the 9 and 10 chemical compounds that satisfied an oral bioavailability (OB) ≥ 10% and drug-likeness (DL) ≥ 0.04 and provide further extraction and optimization of the medicinal ingredients. Nineteen pharmacological ingredients that satisfied the above conditions are showed in Table 1. In a biological network, nodes are visual representations proteins, genes, or metabolites. An edge is a visual representation of a relation. It is a line that connects two nodes and represents biological relationships, such as physical interactions or gene expression regulation [21]. In this study, nodes mean compounds and targets, and edges are relationships between compounds and target. Degree is the number of connected nodes.

To construct a compound-target network, we used Cytoscape 3.6.0 software. By compound-target network, we studied Sangsoo relationships between SR and BR. Multiple targets that participate in 3T3-L1 adipocytes were shared by each compound of SR and BR.

### 2.4. Cell Culture and Adipocyte Differentiation

3T3-L1 preadipocytes (American Type Culture Collection, Rockville, MD, USA) was cultured and differentiated as previously described [22]. Briefly, 3T3-L1 preadipocytes were cultured in DMEM containing 1% penicillin-streptomycin and 10% BS in 6-well plates until confluence at 37°C. Two days after 100% confluence (day 0), cells were differentiated for 48 h in differentiation medium composed of DMEM containing 10% FBS and differentiation inducers (MDI: 0.5 mM IBMX, 1 *μ*M Dex, and 1 *μ*g/ml insulin). From day 2 to day 4, the cells were incubated in culture medium (DMEM plus 10% FBS and 1 *μ*g/ml insulin) supplemented with BR, SR, or BR-SR mixture. Then, at day 4, the medium was changed into fresh culture medium and the cells were cultured for 48 h.

### 2.5. Cell Viability Assay

Cytotoxicity of BR, SR, and BR-SR mixture was evaluated using the MTS assay, as previously described [23]. The absorbance was measured at 490 nm using a VERSAmax microplate reader (Molecular Devices, Sunnyvale, CA, USA).

### 2.6. Oil Red O Staining

Intracellular lipid accumulation was measured by Oil Red O staining as previously described [24]. The absorbance, which is proportional to intracellular lipid, was measured at 500 nm with a VERSAmax microplate reader (Molecular Devices, Sunnyvale, CA, USA).

### 2.7. RNA Isolation and Real-Time Reverse-Transcription Polymerase Chain Reaction (RT-PCR)

Total RNA was extracted from BR, SR, or BR-SR mixture-treated cells using QIAzol lysis reagent (Qiagen Sciences Inc., Germantown, MD, USA) and GeneAll RiboEx Total RNA extraction kit (GeneAll Biotechnology, Seoul, Republic of Korea) as previously described [25]. mRNA evaluation was performed using a StepOnePlus Real-time RT-PCR System (Applied Biosystems, Foster City, CA, USA). Primer sequences used in this study are described in Table 2.

### 2.8. Protein Extraction and Western Blot Analysis

BR, SR, or BR-SR mixture-treated cells were harvested and lysed in Cell Lysis Buffer (Cell Signaling Technology Inc., Danvers, MA, USA). Determination of total protein concentration and western blot analysis were performed as described previously [25, 26].

### 2.9. Statistical Analysis

Results are expressed as mean ± standard error mean (SEM). Student* t*-test was used to determine statistical relevance. All statistical analyses were performed using GraphPad Prism 5 (GraphPad Software, La Jolla, CA, USA).

## 3. Results

### 3.1. Compound-Compound Target Network Analysis of BR and SR

**Figure 1:**
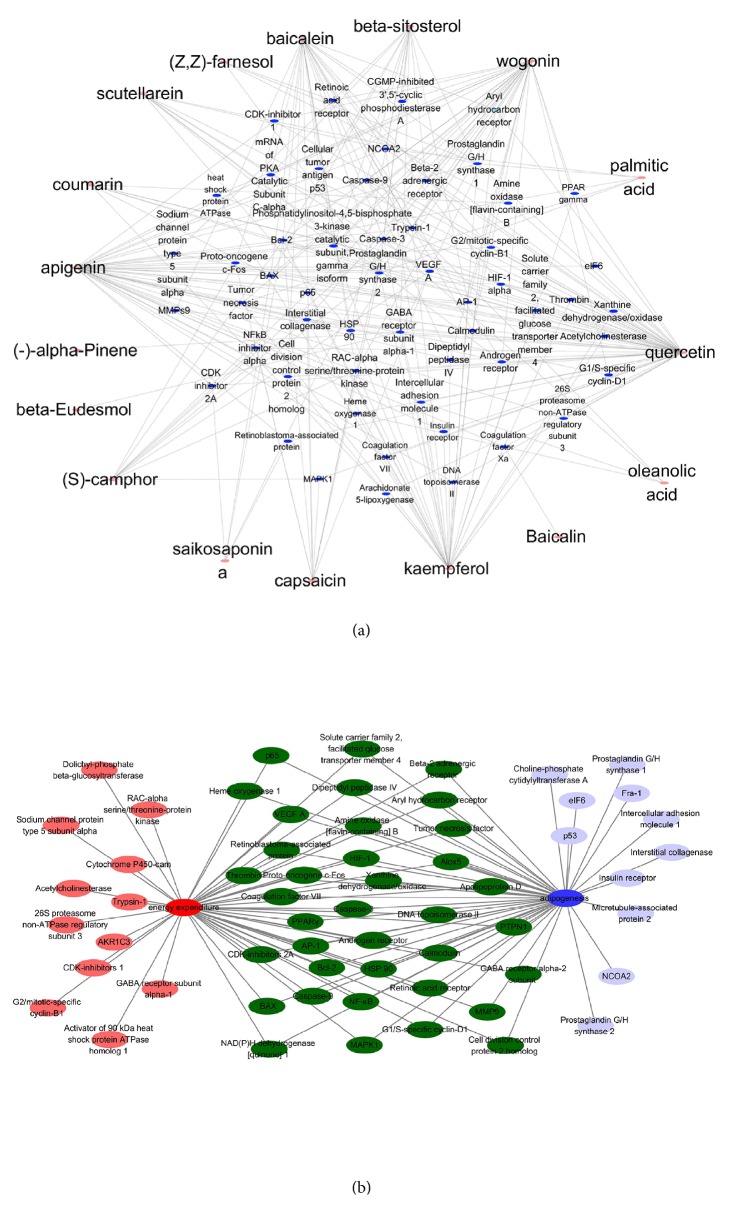
Compound-compound target network of BR and SR. (a) Compound-compound target network of BR and SR. (b) Synergistic target network of BR and SR. Blue ovals represent targets related in adipogenesis, red ovals represent targets related in energy expenditure, and green ovals represent targets related in both.

For prediction analysis of Sangsoo relationship between BR and SR, we constructed the compound-target network (Figure 1(a)) based on data mining. This network consists of 19 compounds and 504 candidate targets in which pink circles and blue ones correspond to active components and targets, respectively. In the relationships between compounds and targets, we found 265 nodes and 504 edges. The means of degree value (the number of associated targets) of the compounds were 18, indicating that most of the compounds regulated multiple targets to exert various therapeutic effects. The mean compounds of each target were 6, showing that most of the targets can interact with multiple compounds simultaneously.

Among the active compounds, quercetin of BR exhibits the highest number of target candidate target interactions (degrees =154), followed by apigenin from SR (degrees = 80), kaempferol from BR (degrees = 59), and wogonin from SR (degrees = 52).

In the 504 targets of active compounds, prostaglandin G/H syntheses 2 showed the highest degree (degrees = 12), which was followed by apoptosis regulator Bcl-2 and caspase-3 (degrees = 10), prostaglandin G/H synthase 1 and phosphatidylinositol-4,5-bisphosphate 3-kinase catalytic subunit, and gamma isoform (degrees = 9). The network pharmacological analysis result demonstrates the potential synergistic effect of SR and BR on adipogenesis and energy expenditure through modulating these relevant proteins.

### 3.2. BR and SR Inhibit Lipid Accumulation in 3T3-L1 Adipocytes

**Figure 2:**
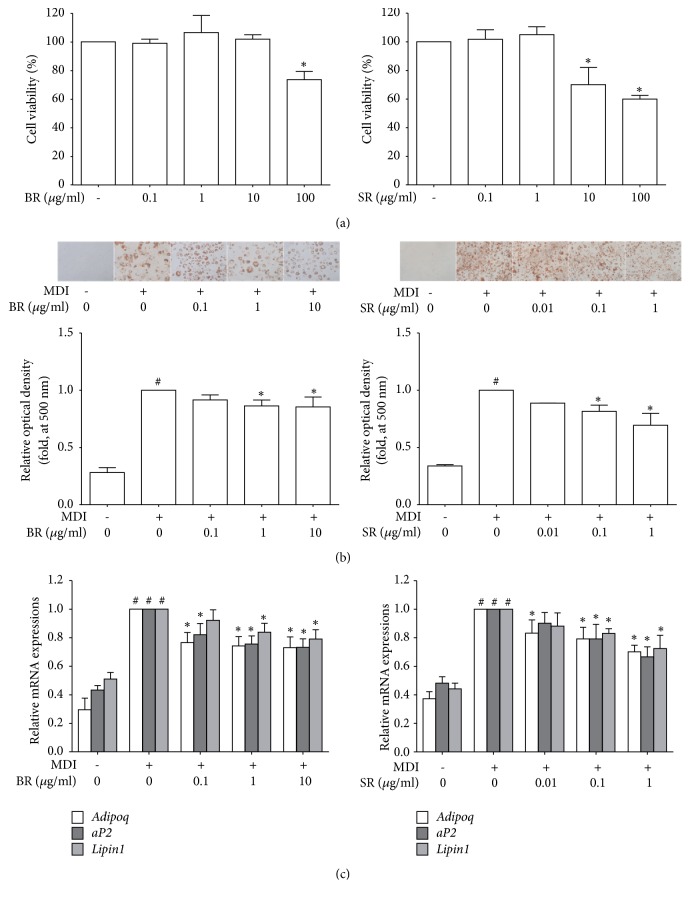
BR and SR inhibit lipid accumulation in 3T3-L1 adipocytes. (a) An MTS assay was performed in order to measure the effects of Bupleuri Radix and Scutellariae Radix on cell viability in 3T3-L1 cells. (b) An Oil Red O assay was performed in order to measure the effect of Bupleuri Radix and Scutellariae Radix on lipid accumulation in 3T3-L1 cells. (c) A Real-Time RT-PCR assay was performed in order to measure the effect of Bupleuri Radix and Scutellariae Radix on mRNA expressions of* AdipoQ*,* aP2,* and* Lipin1*.* Gapdh* mRNA was analyzed as an internal control. Experiments were repeated at least three times. Data represented are the relative expression. All values are mean ± SEM. ^*∗*^*p* < 0.05, significantly different from untreated adipocytes. BR, Bupleuri Radix; SR, Scutellariae Radix.

First, we evaluated the cytotoxicity of BR and SR on 3T3-L1 adipocytes. As shown in Figure 2(a), BR and SR did not affect cell viability up to concentration of 10 *μ*g/ml and 1 *μ*g/ml, respectively, when analyzed by an MTS assay. Further experiments were conducted using concentrations which did not decrease cell viability. Next, to evaluate the effect of BR and SR on lipid accumulation, Oil Red O staining assay was performed. Adipocytes treated with BR and SR both showed concentration-dependent decrease of intracellular lipids (Figure 2(b)).

### 3.3. BR and SR Decrease Adipokines in 3T3-L1 Adipocytes

Real-time RT-PCR assays revealed adipokine genes including* Adipoq*,* aP2*, and* Lipin1* were decreased as well (Figure 2(c)). Adiponectin, an adipokine transcripted by the* Adipoq* gene, promotes adipocyte differentiation by increasing C/EBP*α* and PPAR*γ* in 3T3-L1 adipocytes [27], and also secreted adiponectins are considered as a marker to evaluate adipogenic differentiation [28]. Adipocyte protein 2 (aP2) is a mediator of intracellular transport of fatty acids which is primarily expressed in adipocytes and macrophages [29]. Inhibition of this protein leads to a potential amelioration of obesity [30]. The role of Lipin-1, a protein with the most prominent expression in adipose tissue, skeletal muscle, and testis [31], in adipogenesis is complex. Several studies indicate its importance since the lack of Lipin-1 results in disturbed adipogenesis both* in vivo* and* in vitro* [32–34].

### 3.4. BR and SR Suppress Adipogenesis and Increase Energy Expenditure in 3T3-L1 Adipocytes

**Figure 3:**
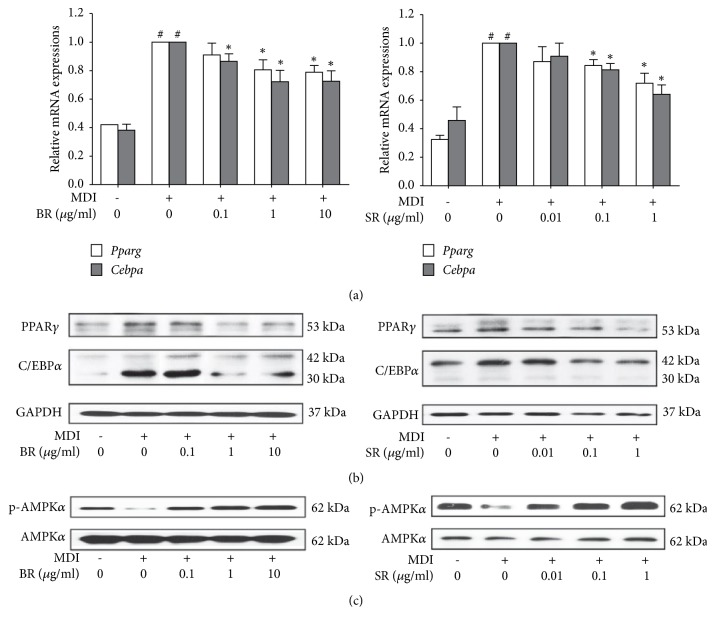
BR and SR suppress adipogenesis and increase energy expenditure in 3T3-L1 adipocytes. (a) A real-time RT-PCR assay was performed in order to measure the effect of Bupleuri Radix and Scutellariae Radix on mRNA expressions of* Cebpa* and* Pparg*. (b) A western blot assay was performed in order to measure the effect of Bupleuri Radix Scutellariae Radix on protein expressions of C/EBP*α*, PPAR*γ*, and (c) p-AMPK*α*.* Gapdh* mRNA was analyzed as an internal control for Real-Time RT-PCR assays. Experiments were repeated at least three times. Data represented are the relative expression. All values are mean ± SEM. ^*∗*^*p* < 0.05, significantly different from untreated adipocytes. BR, Bupleuri Radix; SR, Scutellariae Radix.

To investigate the action mechanism in lipid inhibition by BR and SR, we assessed the change in adipogenic factors. Two major controllers in adipogenesis, C/EBP*α* and PPAR*γ*, were significantly decreased by BR and SR treatment at mRNA level and protein level both (Figures 3(a) and 3(b)), suggesting suppressed adipogenesis was responsible for the antilipogenic effect of BR. Then, we evaluated the effect of BR and SR on AMP-activated protein kinase alpha (AMPK*α*). As a result, we observed concentration-dependent increase in phosphorylation of AMPK*α* in BR- and SR-treated adipocytes (Figure 3(c)). Through these results, we could conclude that BR and SR suppress lipid accumulation in 3T3-L1 adipocytes by two action mechanisms: inhibition of adipogenesis and increase of energy expenditure.

### 3.5. BR-SR Mixture Synergistically Suppress Lipid Accumulation in 3T3-L1 Adipocytes

**Figure 4:**
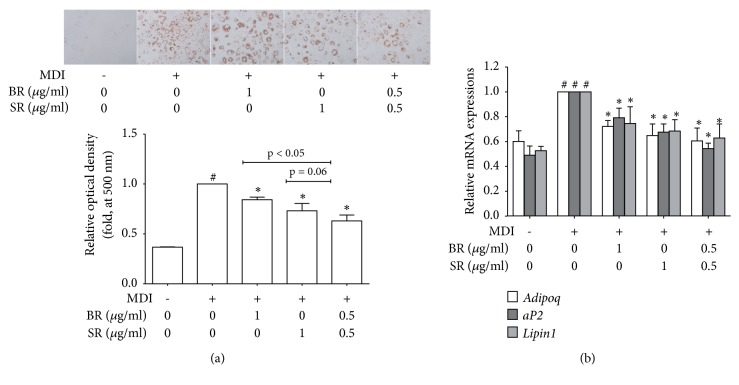
BR-SR mixture synergistically suppresses lipid accumulation in 3T3-L1 adipocytes. (a) An Oil Red O assay was performed in order to measure the effect of Bupleuri Radix, Scutellariae Radix, and their 1:1 mixture on lipid accumulation in 3T3-L1 cells. (b) A Real-Time RT-PCR assay was performed in order to measure the effect of Bupleuri Radix, Scutellariae Radix, and their 1:1 mixture on mRNA expressions of* AdipoQ*,* aP2,* and* Lipin1*.* Gapdh* mRNA was analyzed as an internal control. Experiments were repeated at least three times. Data represented are the relative expression. All values are mean ± SEM. ^*∗*^*p* < 0.05, significantly different from untreated adipocytes. BR, Bupleuri Radix; SR, Scutellariae Radix.

To confirm whether our hypothesis is correct or not, we then performed experiments to evaluate the synergism between BR and SR. BR and SR are contained in Sosiho-Tang at a 3:2 ratio (12 g and 8 g, respectively), and our results on separate extracts showed 1 *μ*g/ml was the most efficient concentration of SR while 1 *μ*g/ml and 10 *μ*g/ml of BR did not differ significantly. Therefore, we used BR-SR mixture at 1 *μ*g/ml of which extracts were mixed at 1:1 ratio. As shown in Figure 4(a), BR-SR 1:1 mixture suppressed lipid accumulation in 3T3-L1 adipocytes, whose inhibition rate was higher than BR 1 *μ*g/ml or SR 1 *μ*g/ml (*p* < 0.05 and* p* = 0.06, respectively). Further qPCR results indicated synergistic inhibition of adipokine genes such as* Adipoq*,* aP2,* and* Lipin1* (Figure 4(b)).

### 3.6. BR-SR Mixture Synergistically Inhibits Adipogenesis and Increases Energy Expenditure

**Figure 5:**
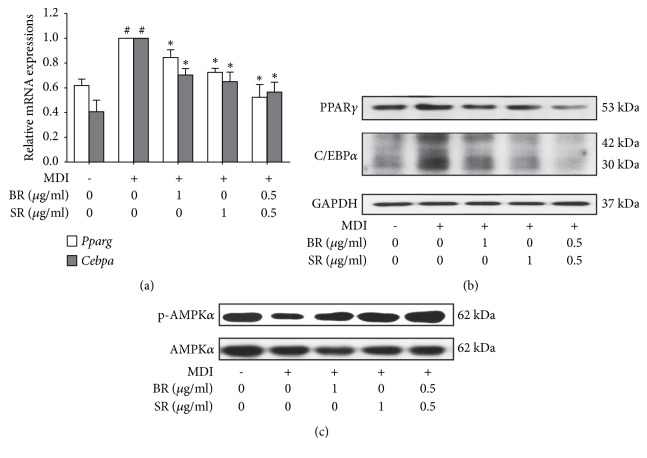
BR-SR mixture synergistically inhibits adipogenesis and increases energy expenditure. (a) A real-time RT-PCR assay was performed in order to measure the effect of Bupleuri Radix, Scutellariae Radix, and their 1:1 mixture on mRNA expressions of* Cebpa* and* Pparg*. (b) A western blot assay was performed in order to measure the effect of Bupleuri Radix, Scutellariae Radix, and their 1:1 mixture on protein expressions of C/EBP*α*, PPAR*γ*, and (c) p-AMPK*α*.* Gapdh* mRNA was analyzed as an internal control for Real-Time RT-PCR assays. Experiments were repeated at least three times. Data represented are the relative expression. All values are mean ± SEM. ^*∗*^*p* < 0.05, significantly different from untreated adipocytes. BR, Bupleuri Radix; SR, Scutellariae Radix.

To investigate the effect of BR-SR mixture on adipogenesis, we analyzed expression levels of* Cebpa* and* Pparg* using Real-Time RT-PCR. As in Figure 5(a), BR-SR 1:1 mixture showed higher inhibition rate in the two factors when compared to separate extracts at the same concentration. Consistently, BR-SR mixture showed synergistic inhibitory effect on protein levels of C/EBP*α* and PPAR*γ* as well (Figure 5(b)). Mixture of BR and SR also enhanced the activation of AMPK*α* (Figure 5(c)), implying that these two herbal extracts synergistically interact in two different pathways of obesity treatment: inhibiting adipogenesis and increasing energy expenditure.

## 4. Discussion

The emerging crisis of obesity impacts the world. A more serious problem is that obesity can lead to other metabolism-related diseases, such as diabetes, cardiovascular diseases, and even cancer [35]. Current available medications for obesity are however limited; therefore the necessity of new options are growing. Nature-derived materials, in this case, have gained interest in the field of obesity treatment due to their positive effects with pharmacological safety [36]. Sosiho-Tang is a Korean medical formula composed of seven herbs: BR (12 g), SR (8 g), Pinelliae Tuber (4 g), Ginseng Radix (4 g), Zizyphi Fructus (4 g), Zingiberis Rhizoma (4 g), and Glycyrrhizae Radix (2 g). This herbal formula is a candidate for optional treatments as Yoo et al. reported its show antiobese effects* in vivo* and* in vitro* [7].

Several studies report the synergistic interaction in nature-derived materials. Our previous study has reported synergistic effect of two herbal pairs,* Vertrum nigrum* and* Panax ginseng* [37]. Zhao et al. used HFD-induced rats to show synergism between quercetin and resveratrol [38]. Another group has also reported the synergistic effect of this pair “browning” of white adipose tissue [39]. In this study, we first analyzed the possible synergy of BR-SR pair by a network pharmacological approach. In the compound-target analysis, we observed several active compounds of BR and SR regulate targets related to adipogenesis and energy expenditure.

Obesity results from accumulation of excess energy in the form of lipid in adipocytes. Adipogenesis is a process of differentiation of preadipocyte into adipocytes. Numerous transcriptional factors form a complex cascade of adipogenesis, of which process changes in shape, genes, proteins, and hormones are accompanied [40, 41]. Among them, C/EBP*α* and PPAR*γ* are considered to be the master regulators in adipogenesis [42]. In the current study, BR-SR mixture showed significant synergism on the inhibition of these two key adipogenic factors.

In addition to inhibiting adipogenesis, pharmacological activation of AMPK*α* is another potential pathway for obesity treatment. The energy storing WAT expresses AMPK, a serine/threonine protein kinase complex composed of three subunits (AMPK*α*, *β* and *γ*) which is activated in low energy-conditions [43]. Activated AMPK induces catalytic ATP generation while inhibiting anabolic process of energy consumption in order to maintain cellular energy homeostasis [44]. Thus, AMPK is one of the key regulators in energy homeostasis that mediate glucose and lipid metabolism to modulate energy levels [45]. Although previous studies allow the anticipation of AMPK elevation by BR [46] and SR [47, 48], the significance of our study was that, by network pharmacological analysis, a possible synergy mechanism on AMPK induction could be expected. Our results show that BR and SR extracts can activate AMPK*α*, suggesting their ability to increase energy expenditure. Furthermore, the 1:1 mixture of these two herbs interacted synergistically, phosphorylating AMPK*α* at a higher level.

Besides energy expenditure, AMPK*α* is also a regulator of adipogenesis by suppressing C/EBP*α* and PPAR*γ* [49]. These reports again provide substantial evidence of synergy between BR and SR on adipogenesis. However, further study is required to fully understand the synergy mechanism of this Sangsoo pair of BR and SR.

## 5. Conclusion

Taken together, we analyzed the network pharmacological action of the Sangsoo herbal pair BR and SR, the two major components of Sosiho-Tang to predict their synergistic possibility. The compound-target relevance showed that these two herbs share two major antiobese targets: adipogenesis and energy expenditure. Then we used 3T3-L1 adipocytes to investigate whether the herbal pair do synergistically interact. As expected, BR-SR mixture showed synergism on suppressing intracellular lipid accumulation and adipokine genes, which resulted from inhibition of adipogenic factors, C/EBP*α* and PPAR*γ*, and activation of the key regulator of energy metabolism, AMPK*α*. Despite the fact that further mechanism study is required and that our results lack in vivo effects of BR-SR herbal pair, our results provide experimental evidence for the Korean medical theory of Sangsoo and also suggest this herbal pair BR and SR may benefit as a potential antiobese therapeutic agent.

## Figures and Tables

**Table 1:** Nineteen active main components of Scutellariae Radix and Bupleuri Radix.

**Scutellariae Radix**	**Bupleuri Radix**
(S)-camphor	(-)-alpha-Pinene
apigenin	(Z,Z)-farnesol
baicalein	Baicalin
Baicalin	beta-Eudesmol
beta-sitosterol	coumarin
scutellarein	kaempferol
palmitic acid	quercetin
wogonin	capsaicin
Linolenic acid methyl ester	saikosaponin a
	oleanolic acid

**Table 2:** Primer sequences (5′ to 3′) for real-time RT-PCR.

Genes	5′ to 3′ Oligonucleotide Sequences
Mouse *Pparg*	
Sense (Forward)	TTT TCA AGG GTG CCA GTT TC
Antisense (Reverse)	TTA TTC ATC AGG GAG GCC AG

Mouse *Cebpa*	
Sense (Forward)	GCC GAG ATA AAG CCA AAC AA
Antisense (Reverse)	CCT TGA CCA AGG AGC TCT CA

Mouse *aP2*	
Sense (Forward)	CGTAAATGGGGATTTGGTCA
Antisense (Reverse)	TCGACTTTCCATCCCACTTC

Mouse *Adipoq*	
Sense (Forward)	AGACCTGGCCACTTTCTCCTCATT
Antisense (Reverse)	AGAGGAACAGGAGAGCTTGCAACA

Mouse *Lipin1*	
Sense (Forward)	TTCCTTGTCCCTGAACTGCT
Antisense (Reverse)	TGAAGACTCGCTGTGAATGG

Mouse *Gapdh*	
Sense (Forward)	AAC TTT GGC ATT GTG GAA GG
Antisense (Reverse)	GGA TGC AGG GAT GAT GTT CT

## Data Availability

The authors will retain all data and can provide it when requested.
